# Can Chemists Turn Wood into an Electronic Material?

**DOI:** 10.1021/acscentsci.3c00949

**Published:** 2023-08-09

**Authors:** Prachi Patel

Forests cover roughly 70% of Sweden, and the sparsely populated country is one of the world’s leading exporters of forestry products. Engineering, meanwhile, is the country’s largest industry. And in one laboratory at Sweden’s KTH Royal Institute of Technology, Jonas Garemark’s PhD research is marrying the two fields.

A video shows six dishes of water sitting on Garemark’s bench, each containing a piece of wood the size of a playing die. When Garemark wires them to a light-emitting diode (LED), the tiny bulb lit up as if connected to a battery.

**Figure:**
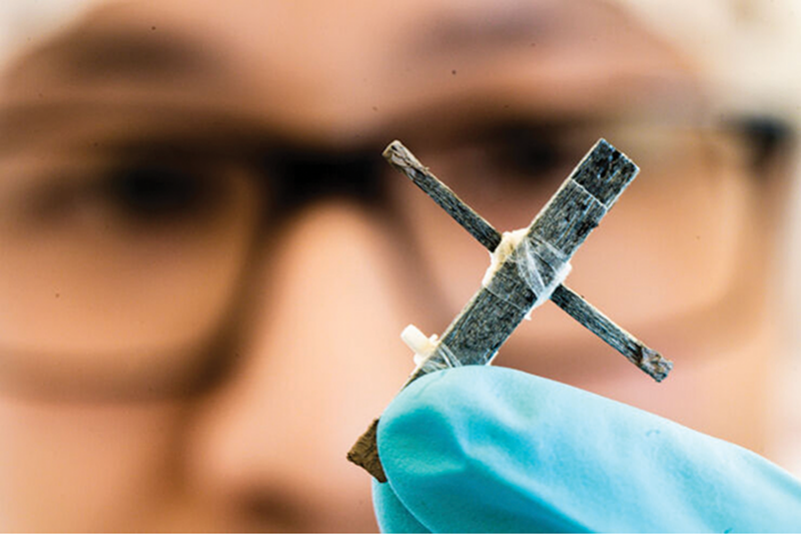
Researchers made the world’s first wooden transistor by removing part of the lignin in wood, filling its microscopic pores with a polymer, and then modulating its conductivity. Credit: Thor Balkhed/Linköping University

While dry wood is an excellent insulator, it can generate small amounts of current when wet. These specially engineered pieces of balsa wood produced 10 times as much electricity as natural wood when the materials were soaked in water, enough to power the LED.

Meter-size pieces could power bigger devices, such as lamps, laptops, and other low-power gadgets in a home. And “you can put wood on even more steroids with additives,” says Yuanyuan Li, a professor of fiber and polymer technology at KTH and Garemark’s PhD adviser. In their most recent work, the researchers impregnated the wood with iron oxide nanoparticles and soaked it to get a power output 165 times that of the original, wet wood.

“As long as it’s in water, the electricity doesn’t stop,” she says. “This can be a feasible battery for low-power devices, especially for remote areas. You could make it at home.”

There are over 3 trillion trees on Earth, making wood one of the planet’s most abundant and renewable resources. Take a close look at this material under the microscope, and it reveals an amazingly intricate hierarchical structure of fibers and pores ranging from a few nanometers to tens of micrometers in size. No matter the tree species, wood is composed of three chemical building blocks in roughly equal parts: the polymers cellulose, hemicellulose, and lignin. These complex biopolymers and their sophisticated arrangement give wood its strength. They also impart lesser-known traits, like the ability to store and transport charge and generate minuscule amounts of electricity.

Li and Garemark—who’s now a postdoctoral researcher at the Swiss Federal Institute of Technology (ETH), Zurich—are part of a small community of researchers who are harnessing and enhancing these properties to transform wood into an electronic material. Using clever chemical engineering to tailor wood’s components and microscopic features, these nanotechnologists have crafted advanced electronic devices, such as energy generators, energy storage devices, and even transistors, the switch-like devices that make logic circuits.

The devices are in their infancy, but they offer a tantalizing vision of an entirely new electronic ecosystem made of wood: a vision of biodegradable environmental sensors; large, wood-based batteries for backup power; and energy-harvesting floors that power lamps using the force from our footsteps.

Substituting even a small amount of plastic and metal with wood would be a big environmental win, says Guido Panzarasa, a chemical scientist in the wood materials science laboratory at ETH Zurich. “Wood is a natural, renewable, widespread, and carbon-sequestering material. Until recently, it was used almost exclusively for structural and decorative purposes. We want to use wood to make electricity, to conduct electricity, and to use electricity.”

## Generation

Liangbing Hu, a materials scientist at the University of Maryland, College Park, embarked on the work that has defined his research when, as a postdoc, he saw microscopy images of nanofibers. These fibers weren’t grown in a lab but forged inside the wood of a tree.

In wood, thousands of such aligned nanofibers form bundles, which are wrapped in hemicellulose and glued together by lignin to form long, micrometers-wide porous fibers. This arrangement of porous fibers, interspersed with tubular channels called lumen, give wood its noble grain. Lumen and the smaller pores in wood fibers are skyscraper elevator shafts, narrow tunnels propelling water up towering tree trunks and distributing it through a network of branches.

What Hu immediately noted upon seeing the cellulose nanofibers, though, was a resemblance to the carbon nanotubes he had been making and studying in the lab. “The tiny fibers had very similar dimensions, and their properties were similar too,” he says. “That’s why I got very excited about this material.”

The images had come from Lars Berglund, a biocomposites researcher at KTH, who had been busting wood apart to liberate these nanofibers. He was studying their outstanding mechanical properties and combining them with other materials to make wood composites.

But Hu had a different idea. Instead of breaking wood apart, why not keep it intact and take advantage of its intricate architecture that has evolved over eons? Removing the lignin, he thought, would keep wood’s cellulose scaffold composed of multiscale pores and channels and would make the cellulose nanofibers accessible for chemical modification.

For decades, the paper industry has removed lignin from wood pulp using acid baths that break down the microstructures in wood. But Hu and his team soak wood in heated chemical baths of basic sodium hydroxide and sodium sulfite, which strip out lignin and leave wood that is flexible and spongy.

One variation on Hu’s process, which involves using high-concentration sodium hydroxide, changes the natural cellulose and forms more hydrogen bonds in the material. The modified cellulose conducts ions better and could lend itself to new applications.

When the researchers soak a thin slice of wood with a sodium hydroxide-based electrolyte and sandwich it between platinum electrodes, a little heat applied to one electrode generates electricity as the sodium ions from the electrolyte move through the cellulose chains. Such devices could scavenge low-level heat from machines or even from the human body to produce power.

Across the ocean, Panzarasa, Ingo Burgert, and their colleagues at ETH Zurich literally squeeze electricity out of the bundles that cellulose nanofibers form in wood. These bundles have highly organized crystalline regions much like those found in materials such as quartz and ceramics. The crystalline domains make all these materials piezoelectric—that is, they generate electricity under mechanical stress because the domains move and shift charges around when the material is deformed.

Native wood, has a much weaker piezoelectric output than quartz because it is stiffer. However, the ETH Zurich team found that making wood spongier by removing lignin boosts piezoelectric output by a factor of more than 55. By connecting nine piezoelectric wood cubes and sandwiching them between two pieces of wood veneer, the researchers made a device about the size of a stick of butter that lights up an LED when pressed.

Meanwhile, the power generator that Li and Garemark’s team made out of wet wood cubes is able to create electricity because of the hydrovoltaic effect: the generation of electricity when materials directly interact with moving water. Wood surfaces have a very slight negative charge. When the wood is wet, positive charges from the dissolved salts and minerals in water build up against these negative charges. As the water evaporates, the movement of dissolved ions generates electricity.

**Figure:**
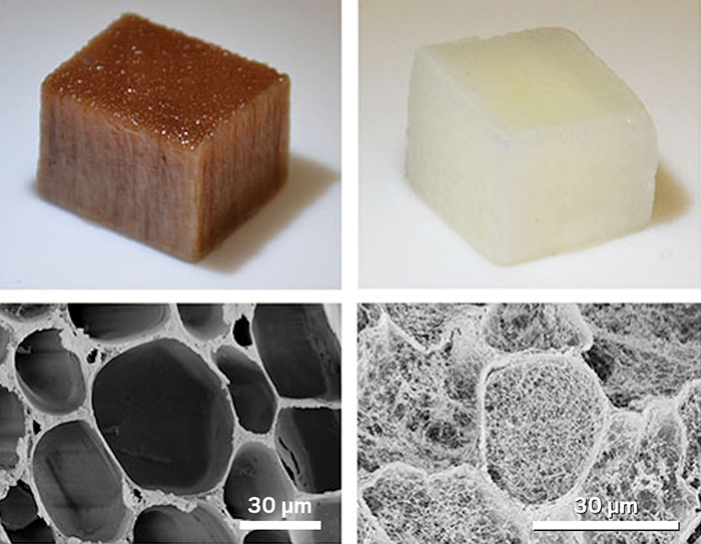
Natural balsa wood (top left) lost its color (top right) after researchers stripped it of its lignin using sodium hydroxide and a freeze-drying process. The treatment also shreds the walls of microscopic channels called lumen, filling the lumen with a tangle of cellulose nanofibers (bottom right). When placed in water, this treated wood produces enough power to turn on a light-emitting diode. Credit: Jonas Garemark.

The researchers boosted the effect 10-fold by soaking balsa wood pieces in a sodium hydroxide solution and then freeze-drying them. That process caused the walls of the wood’s lumen to partially cave in and created a dense tangle of cellulose nanofibers inside the lumen channels. Compared with normal wood, the wood with tangles in the lumen allows for more charge buildup by creating more negatively charged cellulosic surface area. “We can increase surface area 300 times that of natural wood,” Garemark says. In terms of effective area, “instead of a kitchen table, you’d have something as big as a tennis court.”

For all these applications, finding the right tree species for the job is key, Panzarasa says. Woods such as balsa or bass give the best piezoelectric output because their high porosity and strong cellulose structures leave behind highly compressible sponges after they’re delignified. More compressibility means more space for deformation and hence higher piezoelectric output.

Also, because wood comes from trees, there’s some inherent constraints on the shape and size of the material. Hu’s team sometimes cuts wood in a rotary fashion, peeling it spirally and unrolling it to make long, flat pieces. It is “a less-than-ideal material with size limitations and shape complications,” he says. “You have to be really smart about how to deal with it.”

## Conduction

It would seem that these researchers are rebelling against nature: they’re taking wood, a textbook insulator, and trying to conduct electrons through it. Beyond soaking it with water, they are starting to get creative.

Burgert and Panzarasa at ETH Zurich do this by coating wood with iron-gall ink—a blue-black ink made from iron salts and tannic acids that humans have used for writing since antiquity—and blasting it with laser pulses. This converts the tannins and thin layer of underlying wood into a highly conductive, graphite-like carbon material. In a *Nature Communications* paper published last year, they demonstrated the use of this laser-induced graphitized wood to make touch sensors that control light switches as well as humidity and strain sensors.

Another route to conductivity is to simply use wood as a scaffold and fill its holes and cavities with conductive materials. This is the path that Isak Engquist at Linköping University and colleagues took to make the world’s first wooden transistor.

They treat balsa wood to remove some of the lignin and coat the porous wood with a polymer that can be switched between conductive and nonconductive states. Then they sandwich a narrow piece of the polymer-coated wood between two slightly wider pieces of wood in a 3 cm long T shape, putting a dab of gel electrolyte at the cross point.

**Figure:**
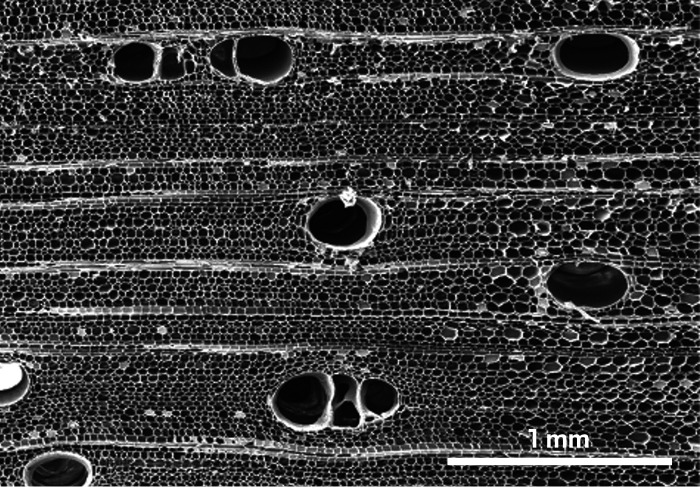
Wood is composed of a parallel arrangement of tubular channels called lumen surrounded by smaller porous fibers. Credit: Liangbing Hu.

When the researchers apply a voltage to the wider pieces, they trigger a chemical reaction that makes the polymer nonconductive. The reaction is reversible, so the researchers can switch the device on and off electronically like a transistor.

Engquist sees wood electronics as a natural evolution from the organic electronics he had long studied. A few years ago, when he got a call from researchers in Berglund’s lab at KTH, it was a matter of “connecting the dots” and seeing the similarities between the properties of wood fibers and the organic conducting polymers he was familiar with, Engquist says. “The conducting polymers perform well when they are configured as a network of fibers, and the wood structure provides a template which offers exactly that.”

## Application

The wood transistor is a lab curiosity for now, and the natural variation in wood means that no two devices are identical. That inconsistency plus the transistor’s size makes it an unlikely choice for any sophisticated electronics. But he says transistors like this could be useful as a simple power switch.

Where wood electronics could really shine, Panzarasa says, is in devices specifically designed not to last, such as environmental or biomedical sensors that safely degrade after use. Aesthetics and simplicity are important too. Wood panels that control devices, wood-based sensors embedded right into structural beams, and energy-generating parquet floors would all be killer apps for wood electronics, he says.

For Maryland’s Hu, an ideal application for wood is sustainable batteries. In lithium-ion batteries, the ions move through the electrolyte from the positive to the negative electrode, and the opposite happens during recharging. But they do so more sluggishly than the electrons that fly through the external wires. That bottleneck limits batteries’ charging speed.

Wood, meanwhile, is also an ionic material, Hu says. “Trees pump water and ions 24/7. Small channels in wood move ions very efficiently. And in batteries, the problem is ions.”

Hu and his team soak delignified wood in copper ion solutions, and as those ions squeeze into the wood, they force open nanometer-scale channels between cellulose nanofibers so that they can transport lithium ions more efficiently. The team is now developing flexible wood-based solid electrolytes and wood-based ion-storing cathodes for lithium-ion batteries.

**Figure:**
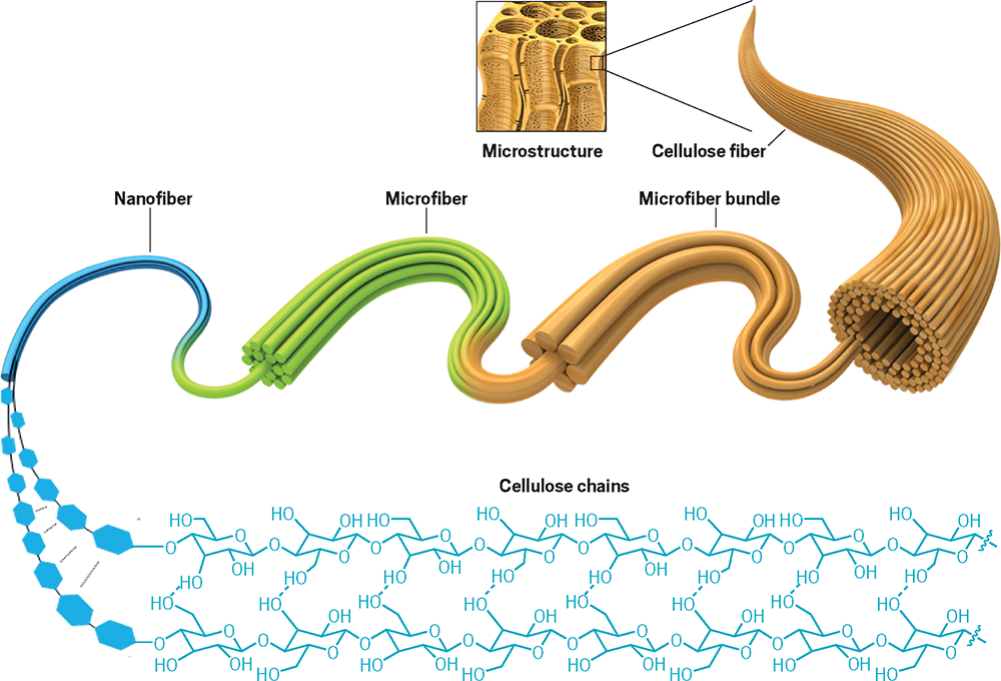
Wood has a complicated hierarchical structure: nanofibers made of cellulose polymers assemble into bundles, which are wrapped in hemicellulose and glued together by lignin to form micrometers-wide porous fibers that compose the bulk of wood. Adapted from *Nat. Mater*.

It’s not likely that such batteries will have the energy density to power an electric car or pocket-sized device. But because their cathodes could eschew costly mined metals like nickel, manganese, and cobalt, wood batteries could present a less expensive and more environmentally friendly option than conventional energy storage. These would be especially suitable for large grid-storage batteries, Hu says. “Wood is not just abundant—it can be better than man-made materials for energy storage if we do it right.”

Besides achieving high performance, doing it right also means maintaining sustainability. Researchers will need to be intentional about their design, using simple, nontoxic chemicals to treat the wood and taking advantage of biodegradable polymers that disappear without a trace along with the rest of the device.

As one option, the ETH Zurich team has come up with a biological approach to remove lignin that relies on white-rot fungi to chew it up. The process is scalable and environmentally friendly, but the downsides are that it is less reliable than chemical delignification and takes weeks rather than hours.

There’s still work to be done, but keeping wood electronics green from the raw material to the final product is on these wood nanoengineers’ minds. “Using green chemistry is very important,” Garemark says. “When you use wood for electronics, you’re using a sustainable, renewable resource, and you don’t want to take away from that.”

*Prachi Patel is a freelance contributor to*Chemical & Engineering News*, the independent news outlet of the American Chemical Society.*

